# Punctate White Matter Lesions Associated With Altered Brain Development And Adverse Motor Outcome In Preterm Infants

**DOI:** 10.1038/s41598-017-13753-x

**Published:** 2017-10-16

**Authors:** Nora Tusor, Manon J. Benders, Serena J. Counsell, Phumza Nongena, Moegamad A. Ederies, Shona Falconer, Andrew Chew, Nuria Gonzalez-Cinca, Joseph V. Hajnal, Sunay Gangadharan, Vasiliki Chatzi, Karina J. Kersbergen, Nigel Kennea, Denis V. Azzopardi, A. David Edwards

**Affiliations:** 1 Centre for the Developing Brain, Perinatal Imaging and Health, Division of Imaging Sciences and Bioengineering, King’s College London, St Thomas’ Hospital, London, SE1 7EH United Kingdom; 2 Department of Neonatology, University Medical Centre Utrecht, Utrecht, The Netherlands; Brain Centre Rudolf Magnus, University Medical Centre Utrecht, Utrecht, 3584 CX The Netherlands; 3 Division of Clinical Sciences, Imperial College London, Hammersmith Hospital, London, W12 0HS United Kingdom; 4 St. George’s, University of London, London, SW17 0QT United Kingdom

## Abstract

Preterm infants who develop neurodevelopmental impairment do not always have recognized abnormalities on cerebral ultrasound, a modality routinely used to assess prognosis. In a high proportion of infants, MRI detects punctate white matter lesions that are not seen on ultrasonography. To determine the relation of punctate lesions to brain development and early neurodevelopmental outcome we used multimodal brain MRI to study a large cohort of preterm infants. Punctate lesions without other focal cerebral or cerebellar lesions were detected at term equivalent age in 123 (24.3%) (59 male) of the 506 infants, predominantly in the centrum semiovale and corona radiata. Infants with lesions had higher gestational age, birth weight, and less chronic lung disease. Punctate lesions showed a dose dependent relation to abnormalities in white matter microstructure, assessed with tract-based spatial statistics, and reduced thalamic volume (p < 0.0001), and predicted unfavourable motor outcome at a median (range) corrected age of 20.2 (18.4–26.3) months with sensitivity (95% confidence intervals) 71 (43–88) and specificity 72 (69–77). Punctate white matter lesions without associated cerebral lesions are common in preterm infants currently not regarded as at highest risk for cerebral injury, and are associated with widespread neuroanatomical abnormalities and adverse early neurodevelopmental outcome.

## Introduction

Preterm infants are at high risk of neurodevelopmental impairment, and cerebral ultrasonography is routinely employed to help assign prognosis and select infants for long-term support. However ultrasound has low sensitivity, and a significant number of infants with normal findings develop motor and cognitive deficits^1,2^; the pathological lesions underlying impairment in these cases are unknown.

In a considerable proportion of preterm infants without other significant brain imaging abnormalities Magnetic Resonance Imaging (MRI) detects punctate white matter lesions (PWML). PWML are highly variable in site and number, can appear at any stage during the perinatal period, typically in more mature infants^3–6^, and may be associated with other lesions^3,7,8^. PWML are suspected to be a manifestation of wider cerebral abnormalities^8–12^, however the clinical importance of PWML, in the absence of other lesions on MRI, remains unclear^7,10,13–18^.

To determine if PWML without other focal lesions might account for neurodevelopmental impairment in preterm infants without other recognised neuroimaging abnormalities we used multimodal MRI data from a prospective multicentre trial of neuroimaging. We defined the incidence, anatomical location and extent of PWML, then tested the hypotheses that: PWML are associated in a dose-dependent manner with abnormal neuroanatomical development, specifically reduced thalamic volume and abnormal white matter microstructure; and that they predict neurodevelopmental impairment at 20 months of age.

## Results

### Participants

**Figure 1:**
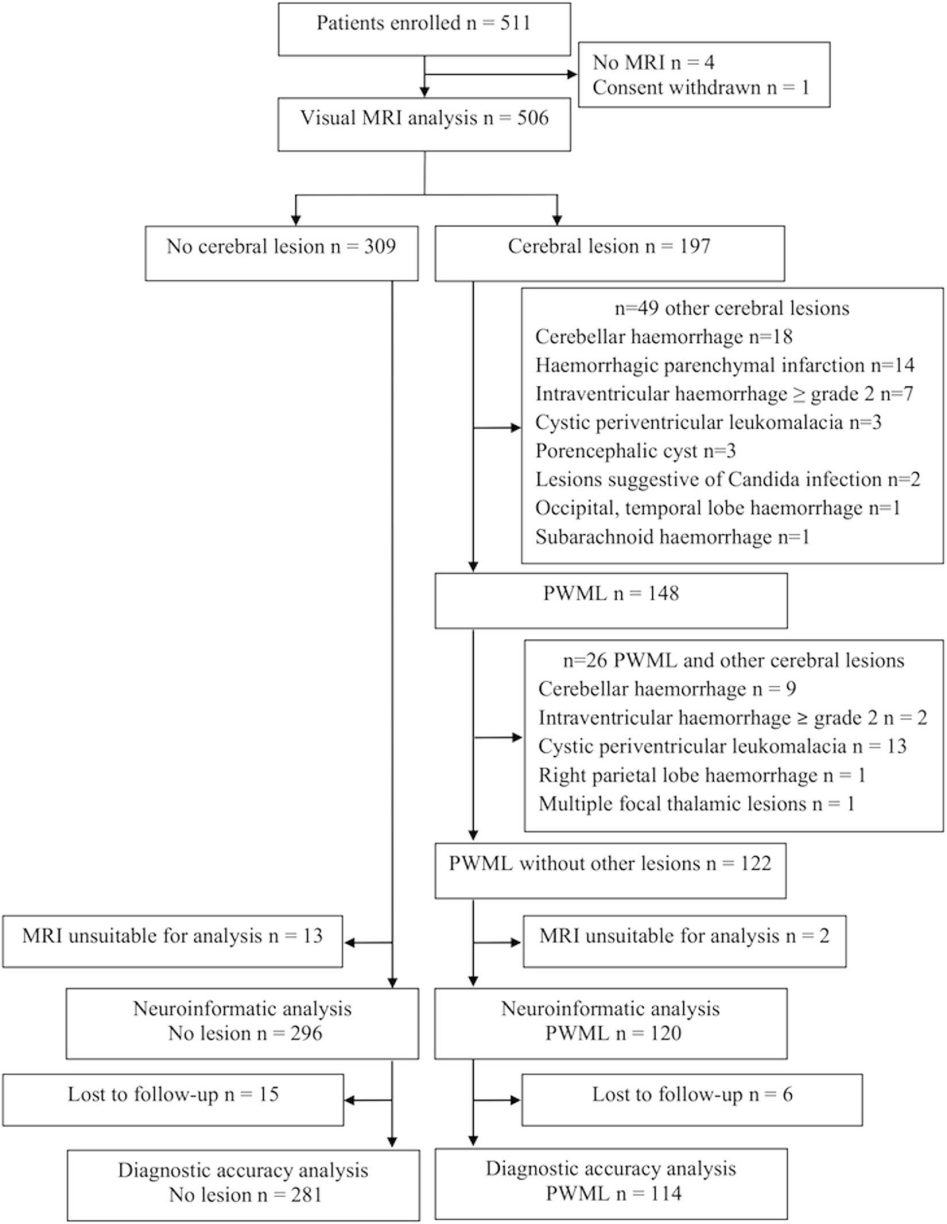
CONSORT diagram. PWML, punctate white matter lesion.

**Figure 2:**
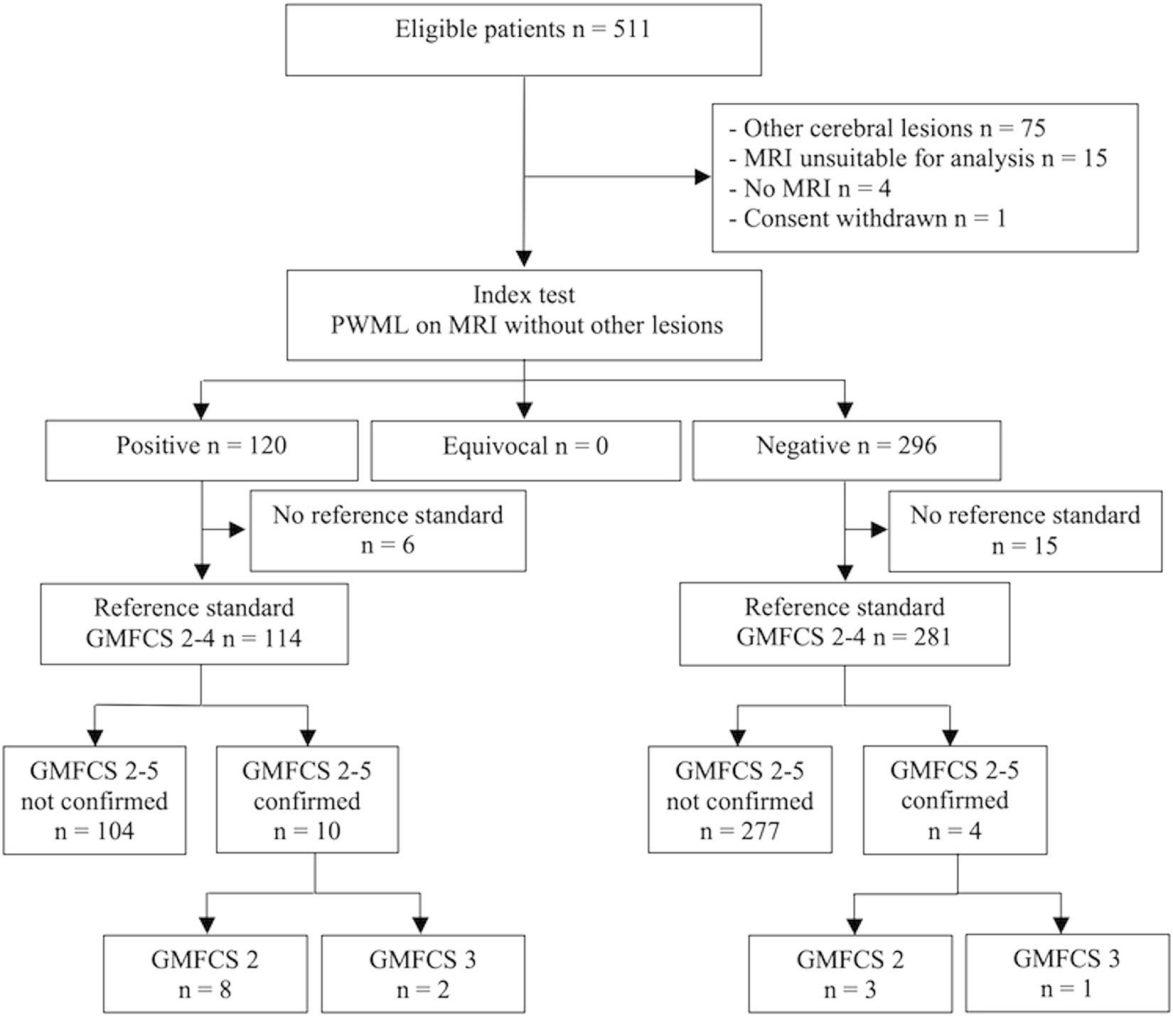
STARD diagram. GMFCS, Gross Motor Classification System; PWML, punctate white matter lesion.

We studied 511 infants (median [range] gestational age 30^+1^ [23^+4^–32^+6^] weeks) who were recruited into a prospective randomised neuroimaging study of preterm infants (Evaluation of Preterm Imaging (ePrime) study). Of the 309 (61%) infants with no PWML or other major focal lesions, 296 (144 male) had appropriate conventional and diffusion tensor imaging (dMRI) data available. Of the 122 (24.1%) infants with PWML 120 (57 male) had suitable MRI data for analysis. This is a secondary analysis of clinical trial data and we report both CONSORT (Fig. 1) and STARD diagrams (Fig. 2).

There was no difference in the rate of clinical chorioamnionitis and prolonged rupture of membranes between infants with no lesions and those with PWML. The rate of maternal urinary tract infection during pregnancy was higher in the PWML group (p = 0.04). Infants with PWML had higher gestational age at birth (p = 0.01) and birth weight (p < 0.001). The duration of invasive (p = 0.03) and non-invasive respiratory support (p < 0.001) was shorter, and the rate of chronic lung disease (p = 0.01), defined as an oxygen requirement at corrected 36 weeks, was lower in the PWML group. Infants with PWML also underwent MRI at an earlier corrected age (p < 0.001) (Table 1).

**Table 1:** Clinical characteristics of infants.

	No lesion n = 296	PWML n = 120	*p*
Age at birth median (range) [weeks]	30^+1^ (23^+4^–32^+6^)	30^+6^ (26^+1^–32^+6^)	0.01
Age at MRI median (range) [weeks]	42^+6^ (37^+6^–58)	42^+3^ (37^+6^–47^+5^)	<0.001
Birth weight median (range) [kg]	1.22 (0.57–2.6)	1.47 (0.74–2.51)	<0.001
Male n (%)	144 (48.6)	57 (47.5)	0.8
Multiple pregnancy n (%)	99 (33.4)	42 (35)	0.8
Prolonged rupture of membranes n (%)	42 (14.2%)	23 (19.2%)	0.13
Clinical chorioamnionitis n (%)	16 (5.4%)	6 (5%)	0.54
Maternal UTI during pregnancy n (%)	4 (1.4%)	6 (5%)	0.04
No antenatal steroids n (%)	9 (3)	4 (3.3)	0.8
Administration of surfactant n (%)	156 (52.7%)	57 (47.5%)	0.20
Days on invasive ventilation mean (SD)	2.81 (6.3)	1.68 (4.29)	0.03
Days on non-invasive ventilation mean (SD)	17.81 (20.36)	10.68 (14.67)	<0.001
Chronic lung disease n (%)	N = 269 80 (29.7)	N = 110 19 (17.2)	0.01
Days on parenteral nutrition mean (SD)	8.36 (10.93)	6.63 (11.29)	0.15
Any treatment for PDA n (%)	16 (5.4%)	6 (5%)	0.54
Surgical treatment of NEC n (%)	6 (2%)	1 (0.8%)	0.35
Multiple deprivation score mean (SD)	20.57 (11.9)	20.03 (11.7)	0.6

PDA, patent ductus arteriosus; NEC, necrotizing enterocolitis; PWML, punctate white matter lesions; SD, standard deviation; UTI, urinary tract infection.

### Relation between PWML and neuroanatomical abnormalities

**Figure 3:**
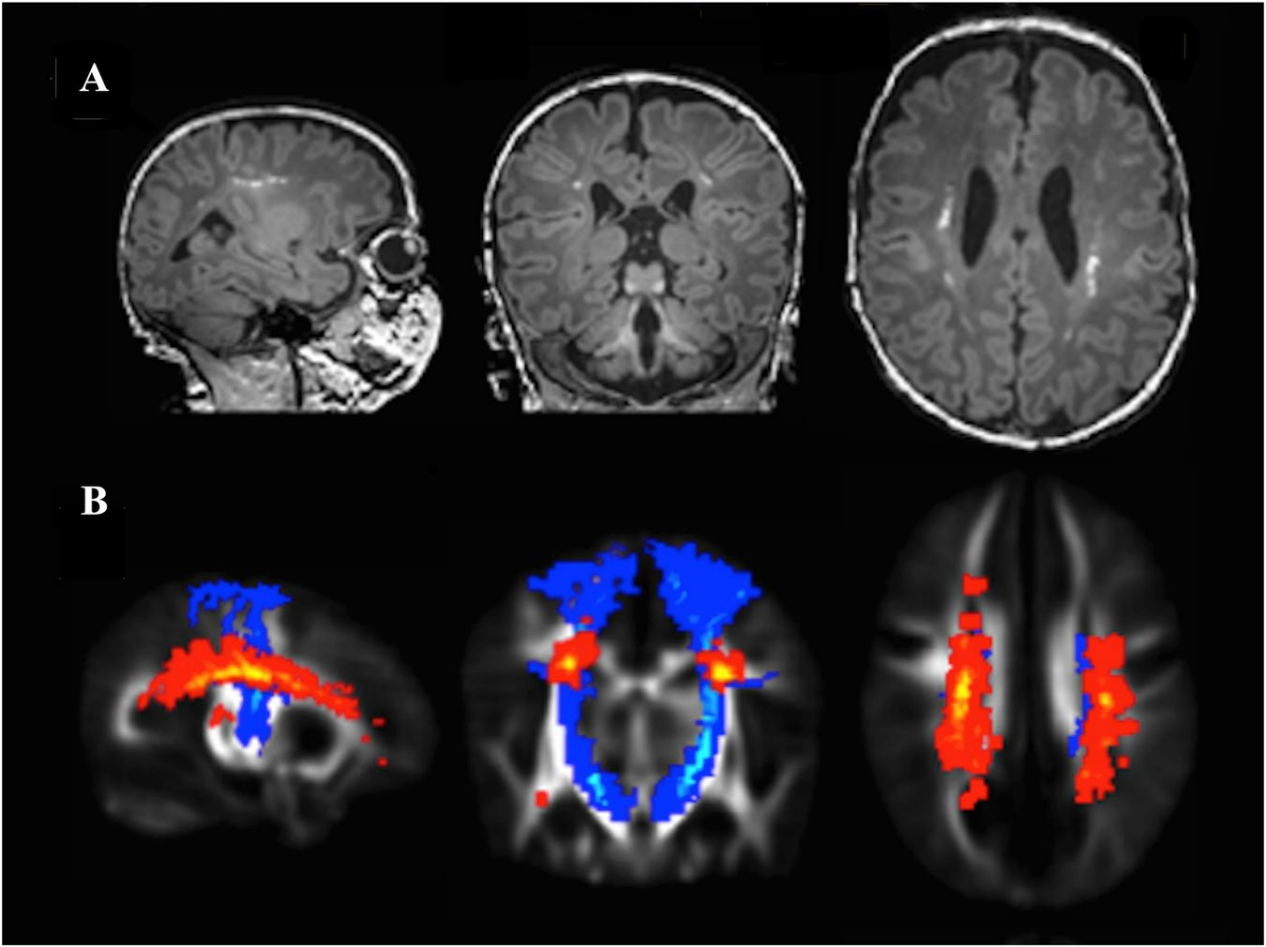
Typical location and distribution of punctate white matter lesions. (**A**) Punctate white matter lesions apparent as high signal focal lesions on magnetization-prepared rapid gradient echo image in a single infant. (**B**) Group-level probabilistic lesion map (yellow-red) in relation to the corticospinal tracts (blue) overlaid on a 40-week T2-weighted template. Images are displayed in sagittal, coronal and transverse views.

PWML were predominantly seen in the regions of the centrum semiovale, corona radiata, and arcuate fasciculi. Median (range) absolute PWML volume was 0.03 (0.001–0.89) cm^3^. 16 (13.3%) infants had more than 20 PWML, and 85 (70.8%) had lesions in the regions of the corticospinal tracts. Examples of PWML in a single infant and the group level probabilistic map of lesion incidence and location in relation to the corticospinal tracts are shown in Fig. 3 and in Supplementary video S1.

Mean (standard deviation (SD)) total brain volumes were comparable between infants with no lesions (381.23 (47.4) cm^3^) and those with PWML (381.91 (44.3) cm^3^) (p = 0.8). Mean (SD) absolute and relative thalamic volumes were significantly smaller (p < 0.001, partial eta squared η_p_^2^ = 0.44 and η_p_^2^ = 0.16 respectively) in the PWML group (5.27 (0.8) cm^3^ and (0.014 (0.001)) compared to the no lesion group (5.71 (0.8) cm^3^ and (0.015 (0.001)). There was a significant negative correlation between lesion load and thalamic volume (absolute: R^2^ = 0.22 p < 0.001; relative: R^2^ = 0.26 p < 0.001).

**Figure 4:**
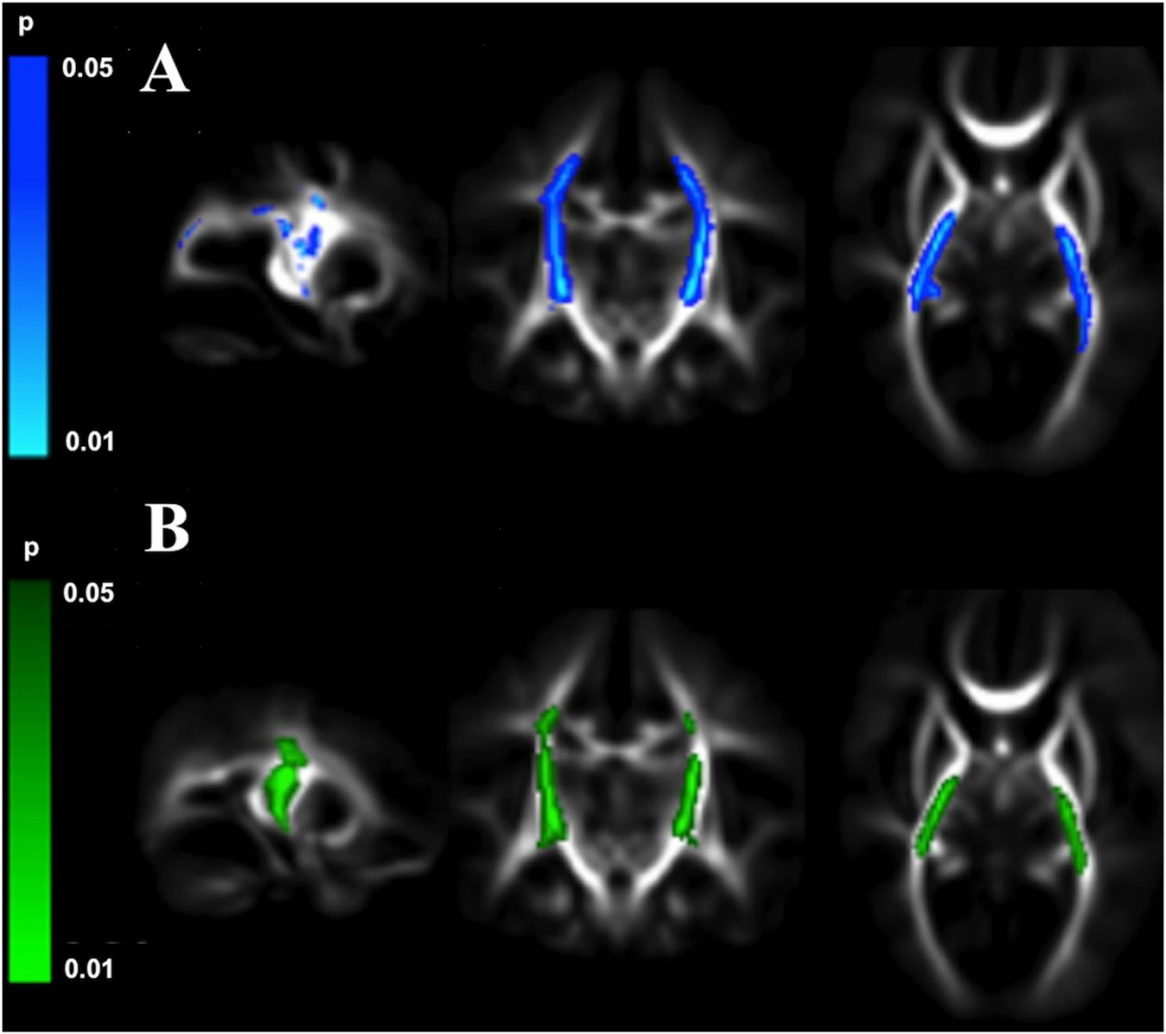
Punctate white matter lesions are associated with altered white matter microstructure. Mean fractional anisotropy maps in sagittal, coronal, and transverse views showing white matter regions where infants with punctate lesions had significantly (**A**). higher radial diffusivity (blue) and (**B**). lower fractional anisotropy (green) as assessed with tract-based spatial statistics.

Significantly lower fractional anisotropy and higher radial diffusivity values were found in the centrum semiovale, corona radiata, posterior limb of the internal capsule, and arcuate fasciculi in infants with PWML (Fig. 4). Many but not all of these brain regions intersect with the locations of PWML. Greater lesion load was associated with higher radial diffusivity (R^2^ = 0.10 p < 0.001) and lower fractional anisotropy (R^2^ = 0.11 p < 0.001) in regions of the corona radiata and posterior limb of internal capsule.

### Relation between PWML and neurodevelopmental outcome

Neurodevelopmental assessments were performed at a median (range) corrected 20.2 (18.4–29.3) months on 281 children with no lesions and 114 with PWML on MRI at term equivalent age. Moderate or severe functional motor disability was more common amongst infants with PWML (n = 10 (8.7%)) compared to those with no lesions (n = 4 (1.4%)) (p < 0.001). Larger lesion load correlated with higher Gross Motor Function Classification System (GMFCS)^19,20^ level (p < 0.001; η_p_^2^ = 0.82), and lower Bayley Scales of Infant and Toddler Development®, third edition (BSID-III)^21^ motor scores (p < 0.001; η_p_^2^ = 0.86), but not with language (p = 0.07) or cognitive (p = 0.25) scores. Predictive values for motor outcomes are given in Table 2 for any PWML (n = 114); PWML in the corticospinal tracts (n = 80); and more than 20 PWML on MRI (n = 16). Higher lesion load predicted functional motor impairment more precisely. The sensitivity analysis for number of PWML showed that a cut-off of 20 lesions was more predictive than previously reported lower cut-offs (Table 3)^10,13–16^.

**Table 2:** Predictive values (with 95% confidence intervals) of punctate lesions to estimate motor outcome with presence of any punctate lesions, punctate lesions in the corticospinal tracts, and more than 20 punctate lesions on conventional MR images.

Outcome measure	Presence of any PWML		Any PWML in corticospinal tracts		>20 PWML on MRI	
	GMFCS 2–5	Motor score <85	GMFCS 2–5	Motor score <85	GMFCS 2–5	Motor score <85
Sensitivity	0.71 (0.42–0.92)	0.33 (0.2–0.49)	0.64 (0.35–0.87)	0.31 (0.18–0.47)	0.5 (0.23–0.77)	0.18 (0.08–0.32)
Specificity	0.73 (0.68–0.77)	0.72 (0.67–0.76)	0.82 (0.77–0.85)	0.81 (0.77–0.85)	0.98 (0.96–0.99)	0.98 (0.96–0.99)
Positive predictive value	0.09 (0.04–0.15)	0.13 (0.08–0.2)	0.11 (0.05–0.2)	0.18 (0.1–0.28)	0.44 (0.2–0.7)	0.5 (0.25–0.75)
Negative predictive value	0.98 (0.96–0.99)	0.89 (0.85–0.93)	0.96 (0.96–0.99)	0.9 (0.8–0.93)	0.98 (0.96–0.99)	0.9 (0.87–0.93)
Positive likelihood ratio	2.62 (1.81–3.79)	1.18 (0.75–1.15)	3. 5 (2.24–5.46)	1.68 (1–2.7)	21.17 (9.22–48.6)	7.78 (3.97–19.7)
Negative likelihood ratio	0.39 (0.17–0.90)	0.93 (0.75–1.15)	0.44 (0.22–0.88)	0.85 (0.69–1.04)	0.51 (0.3–0.86)	0.84 (0.73–0.96)
Diagnostic odds ratio	6.65 (2.04–21.7)	1.26 (0.65–2.46)	7.99 (2.6–24.6)	1.98 (0.99–3.93)	41.3 (11.9–142.7)	9.24 (3.28–26.07)

GMFCS, Gross Motor Function Classification System; PWML, punctate white matter lesions.

**Table 3:** Predictive values (with 95% confidence intervals) of punctate lesions to estimate motor outcome with different numerical cut-offs for lesion load.

Outcome measure	>3 PWML on MRI		>6 PWML on MRI		>20 PWML on MRI	
	GMFCS 2–5	Motor score <85	GMFCS 2–5	Motor score <85	GMFCS 2–5	Motor score <85
Sensitivity	0.57 (0.32–0.78)	0.29 (0.18–0.43)	0.57 (0.33–0.79)	0.24 (0.13–0.39)	0.57 (0.33–0.79)	0.22 (0.13–0.36)
Specificity	0.85 (0.81–0.89)	0.85 (0.81–0.89)	0.9 (0.87–0.93)	0.9 (0.86–0.93)	0.94 (0.91–0.96)	0.94 (0.91–0.96)
Positive predictive value	0.13 (0.06–0.23)	0.2 (0.11–0.32)	0.17 (0.08–31.4)	0.24 (0.13–0.39)	0.27 (0.12–0.46)	0.33 (0.17–0.53)
Negative predictive value	0.98 (0.96–0.99)	0.9 (0.87–0.93)	0.98 (0.96–0.99)	0.9 (0.87–0.93)	0.98 (0.97–0.99)	0.9 (0.87–0.93)
Positive likelihood ratio	3.89 (2.32–6.5)	1.98 (1.17–3.35)	5.73 (3.32–9.88)	2.44 (1.34–4.46)	9.9 (5.36–18.19)	3.89 (1.95–7.78)
Negative likelihood ratio	0.5 (0.27–0.92)	0.83 (0.69–1)	0.48 (0.26–0.87)	0.84 (0.71–0.99)	0.46 (0.25–0.83)	0.82 (0.7–0.97)
Diagnostic odds ratio	7.74 (2.59–23.15)	2.38 (1.17–4.84)	12.03 (3.97–36.53)	2.91 (1.36–6.25)	21.76 (6.94–68.21)	4.71 (2.1–10.87)

GMFCS, Gross Motor Function Classification System; PWML, punctate white matter lesions.

### Relation to cerebral ultrasound imaging

No PWML were detected by ultrasound for any of the infants. Review of previous ultrasound reports showed two infants with evidence of cystic periventricular leukomalacia which was not apparent on MRI at term equivalent age. One infant had PWML at term equivalent age and the other did not.

## Discussion

PWML detected on a single MRI at term equivalent age without other focal lesions or injuries in the grey matter were associated with abnormal neuroanatomical development and adverse motor outcome at 20 months. The effect on both structure and function was dose-dependent. As a predictor of impaired early motor outcome, PWML had a sensitivity similar to previous studies which have assigned prognosis based on analysis of all MR features, but ignoring the presence of PWML^22^. PWML thus appear to represent a neuropathological process which has implications for neurodevelopment.

PWML were common, and present in almost one-quarter of infants, which is consistent with previous smaller studies^7,10,13,14^. The lesions occurred frequently in more mature infants with larger birth weight thought to be at lower risk of long-term adverse consequences^3,5,6^. This suggests that the current approaches to assigning increased risk, which are based predominantly on lower gestational age and abnormal ultrasound images, are insensitive for predicting PWML and thus abnormal brain development and outcome.

PWML were distributed widely in the centrum semiovale and corona radiata but, similar to a previous report^17^, they were more common in the areas traversed by the corticospinal tracts. The strong prediction of motor impairment in children with PWML is in line with previous smaller studies that reported a relationship between PWML and delayed motor development^10,14,16^ or cerebral palsy^14,15,17^. The correlation between PWML, particularly in the corticospinal tract and motor deficits suggest that the anatomical location of lesions is significant; indeed, a recent study in which PWML were predominantly seen in the frontal regions found them to be predictive of adverse cognitive outcome at 18 months^18^. This information is of value for neuroradiological interpretation. However, neuroinformatic analysis showed that PWML were associated with widespread cerebral abnormalities, including altered white matter microstructure in regions where the lesions were not apparent visually, and reduced relative thalamic volume. PWML are thus associated with more widespread abnormalities than is observed by MRI^9–11^. It is possible that while the apparent relation to motor deficits represents the higher incidence of corticospinal tract lesions in our cohort, as well as the relative sensitivity of neurodevelopmental testing to motor problems at around 20 months. However, as the children grow older other deficits may come to light associated with these more widespread effects, and involving other brain networks, such as the afferent thalamocortical radiations^23^.

To assess neurodevelopmental outcomes we employed commonly used tests, selecting moderate or severe functional motor impairment as a clinically relevant endpoint^19,20,24^. The GMFCS has been used successfully before in preterm infants, and reduces the variability inherent in separating mild impairment from typical development at this age^25^. It has been suggested that GMFCS is less stable before the age of two^20^, however a high lesion load also predicted suspected motor impairment defined by the BSID-III, an established test which correlates with, although probably underestimates, long-term deficits^26^. Both the GMFCS^7,14^ and BSID-III^10,27^ were used previously to explore the prognostic significance of PWML.

In conclusion, these results suggest that infants with PWML, particularly if the lesions involve the corticospinal tracts and if there are more than 20, should be recognized as being at high risk for later adverse outcomes, and that the neuropathological processes underlying PWML may help explain the significant proportion of preterm children with cerebral palsy who have a normal cerebral ultrasound at term equivalent age^1^. It is now important to understand these processes better.

## Methods

We studied infants recruited into a prospective randomised neuroimaging study of preterm infants (ePrime study) between 2010 and 2013 from 14 neonatal units (level 1, 2 and 3) in London who were imaged at term equivalent age at a specialist Neonatal Imaging Centre. Infants were eligible if born before 33 weeks gestational age and their mother was aged over 16 years and not a hospital inpatient; they were excluded if they had congenital malformation, prior MRI, care in a centre where preterm MRI was routine, non-MRI compatible implants, non English-speaking parents, or were subject to child protection proceedings. As this study was a secondary analysis the trial sample size was not based on an estimate of the predictive power of PWML.

Clinical and demographic data were collected from the hospital records and discharge letters. An index of socioeconomic status was calculated based on the postal district of the mother’s address^28^. Data were checked for normality, and appropriate parametric or non-parametric tests were applied to compare clinical and demographic characteristics of infants with PWML to those with no lesions.

### Standard Protocol Approvals, Registrations and Patient Consents

The Hammersmith, Queen Charlotte’s and Chelsea Research Ethics Committee reviewed and approved the protocol (09/H0707/98). Written informed consent was obtained from the parent or guardian in every case. The ePrime study was registered with the European Clinical Trials (EudraCT-2009-011602-42) and Clinical. Trials.gov (NCT01049594) on 13/01/2010 before recruitment began. The study was overseen by an independent steering committee with advice from a data monitoring and ethics committee. The study was conducted in accordance with the recommendations for physicians involved in research on human subjects adopted by the 18^th^ World Medical Assembly, Helsinki 1964 and later revisions. Datasets generated and analysed during the current study are available from the corresponding author on request.

### Magnetic Resonance Imaging

Infants underwent brain MRI at term equivalent age on a 3-Tesla system. Parents were offered the choice of having their infants sedated with chloral hydrate. Physiological parameters (heart rate, oxygen saturation, and temperature) were monitored before and during the scan by a pediatrician experienced in MRI procedures. Each infant’s head and body were gently restrained during scanning using a vacuum evacuated polyester system, with the infants closely swaddled and ear protection used to minimize scanner noise disturbing the infant’s sleep. Ear protection comprised of individually moulded earplugs made of silicone-based putty (President Putty; Coltene/Whaledent, Mahwah, NJ) placed into the external ear, and neonatal earmuffs (Natus MiniMuffs; Natus Medical, San Carlos, CA).

The imaging protocol included 3D magnetization-prepared rapid gradient echo (MP-RAGE) (repetition time (TR) = 17 ms, echo time (TE) = 4.6 ms, flip angle 13°, slice thickness 0.8 mm, voxel size: 0.82 × 0.82), T2-weighted turbo spin echo (TR = 8670 ms, TE = 160 ms, flip angle 90°, slice thickness 2 mm with 1mm overlapping slices, in-plane resolution 0.86 × 0.86 mm), and single-shot echo-planar dMRI sequences with a single *b* = 0 volume (TR = 7536 ms; TE = 49 ms; flip angle 90°; slice thickness 2 mm; in plane resolution 2 × 2 mm, 32 non-collinear gradient directions, *b* value of 750 s/mm^2^).

Conventional images were inspected for motion artifacts and subjects with gross motion were excluded. Diffusion MRI volumes were also inspected visually to detect volumes with motion artifact. All infants included in the study had at most 8 gradient directions excluded from the diffusion data. Two subjects with punctate lesion and 13 with no lesion were excluded from the analysis due to motion artifact on MRI.

Conventional images were inspected to define PWML, apparent as high signal focal lesions on MP-RAGE images, and other cerebral lesions. Infants with major focal cerebellar or cerebral injury, including periventricular leukomalacia, intraventricular haemorrhage grade two or more, porencephalic cyst, haemorrhagic parenchymal infarction, and those whose MRI data were not suitable for analysis because of insufficient quality due to motion were excluded.

### Image analysis

All images were analysed using tools implemented in Functional MRI of the Brain Software Library^29^ and Image Registration Toolkit^30^ by authors unaware of the clinical history and neurodevelopmental outcome.

### Probabilistic lesion map

For each infant with PWML, a binary lesion mask was created manually on the MP-RAGE and propagated to the T2-weighted image. To ascertain the spatial extent and location of PWML at the group level a probabilistic lesion map was generated by propagating each mask from the individual T2-weighted image to a 40-week neonatal template. Individual template-space lesion maps were inspected in relation to the corticospinal tracts defined by diffusion tractography, and infants were divided into two groups based on whether they had PWML along the corticospinal tracts or elsewhere.

### Volumetric analysis

For each infant binary thalamic and whole brain masks were derived with automated tissue segmentation driven by age-specific priors^31^. Absolute total brain, thalamic and PWML volumes were determined. Relative volumes were calculated by dividing absolute thalamic and PWML volumes respectively by total brain volume. Total brain, absolute and relative thalamic volumes were compared between infants with no lesions and those with PWML using analysis of variance. Within the PWML group the correlation between lesion load and thalamic volume was assessed by linear regression. In addition to lesion volume, the number of PWML was estimated by inspection, and infants divided into two groups based on whether they had more or fewer than 20 PWML.

### Diffusion Imaging

dMRI data were pre-processed using Diffusion Toolkit^32^. Diffusion weighted volumes were corrected for echo planar imaging phase encoding distortions, eddy-current induced distortions and subject movement by aligning all diffusion volumes to the reference *b* = 0 image, and the corresponding *b*-vectors rotated accordingly. Fractional anisotropy maps were created, and radial diffusivity maps were derived after averaging the 2^nd^ and 3^rd^ eigenvalues. Voxel-wise pre-processing of fractional anisotropy data on a group level was carried out using a tract-based spatial statistics protocol that was optimised for the neonatal brain^33^. Every infant’s fractional anisotropy map was aligned to a chosen target to create a mean fractional anisotropy map and skeleton. Each infant’s aligned fractional anisotropy map was projected onto the mean fractional anisotropy skeleton and the nonlinear warps and skeleton projections were applied to the radial diffusivity maps.

To test whether infants with PWML had altered white matter microstructure we used voxel-wise non-parametric permutation testing^34^ to compare fractional anisotropy and radial diffusivity between infants with and without PWML, and to assess the relation of these measures to lesion load. Results were corrected for multiple comparisons voxel-wise by controlling family-wise error rate after threshold-free cluster enhancement and p < 0.05 was considered significant.

### Relation to cerebral ultrasound imaging

Infants were examined by cerebral ultrasound on the same day as the MRI. To assess whether there were transient ultrasound changes that had resolved by term equivalent age clinical ultrasound reports were reviewed.

### Neurodevelopmental assessment

Infants were assessed at corrected 20 months between 2012 and 2015 by a developmental psychologist unaware of the clinical history and neuroimaging findings. The principal reference standard for an unfavourable outcome was moderate or severe functional motor impairment, defined as GMFCS grade 2–5. We supported this by exploring the prediction of probable motor, cognitive or language impairment defined as a score of more than one standard deviation below the mean on the BSID-III.

### Correlation of MR imaging to outcome

Linear regression was used to assess the relation of PWML load to GMFCS level and BSID-III scores. Where a significant correlation was found between lesion load and outcome measures, the predictive value of any PWML, PWML in the corticospinal tract and the presence of more than 20 PWML was calculated after dividing the infants into two groups of favourable and unfavourable outcome. In a sensitivity analysis the predictive value of different previously reported numerical cut-offs for number of lesions was also tested^10,13–16^. Predictive values with 95% confidence intervals were calculated from 2 × 2 contingency tables. All estimations were made using SPSS 23.0 (SPSS Inc., Chicago, IL, USA).

## Electronic supplementary material

Supplementary video S1

Supplementary information file
