# Supplementary material for: Punctate White Matter Lesions Associated With Altered Brain Development And Adverse Motor Outcome In Preterm Infants
Source: Sci Rep. 2017 Oct 16;7:13250. doi: 10.1038/s41598-017-13753-x (PMC5643493; doi:10.1038/s41598-017-13753-x)
Supplement: Supplementary file 2 — Supplementary information file [file 41598_2017_13753_MOESM2_ESM.pdf]

Punctate White Matter Lesions Associated With Altered Brain Development And Adverse Motor Outcome In Preterm Infants

Nora Tusor[1*], Manon J. Benders[2], Serena J. Counsell[1], Phumza Nongena[3], Moegamad A Ederies[3], Shona Falconer[1], Andrew Chew[1], Nuria Gonzalez-Cinca[1], Joseph V. Hajnal[1], Sunay Gangadharan[1], Vasiliki Chatzi[1], Karina J. Kersbergen[2], Nigel Kennea[4], Denis V. Azzopardi[1], A. David Edwards[1]

Supplementary video S1 online: Group level probabilistic punctate white matter lesion map in relation to the corticospinal tracts. 3D movie of the group level probabilistic punctate lesion map (yellow-red) in relation to the corticospinal tracts (blue) displayed on a 40-week T2-weighted neonatal template as in Figure 3.
